# Prevalence and predictors of preconception medical and behavioral risks among soon-to-be married couples: A quantitative cross-sectional survey in Rwanda

**DOI:** 10.1371/journal.pone.0336023

**Published:** 2026-01-16

**Authors:** Richard Nsengiyumva, Thiery Claudien Uhawenimana, Olive Bazirete, Darius Gishoma, Carlsson Ing-Marie, Lee HaEun, Jody Rae Lori

**Affiliations:** 1 School of Nursing and Midwifery, University of Rwanda, Kigali, Rwanda; 2 Mental Health Division, Rwanda Biomedical Center, Ministry of Health Rwanda, Kigali, Rwanda; 3 School of Nursing and Midwifery, Halmstad University, Halmstad, Sweden; 4 Department of Systems, Population, and Leadership, School of Nursing, University of Michigan, Ann Arbor, Michigan, United States of America; 5 Department of Health Behavior and Biological Sciences, School of Nursing, University of Michigan, Ann Arbor, Michigan, United States of America; University of North Carolina at Greensboro, UNITED STATES OF AMERICA

## Abstract

**Background:**

Maternal and neonatal morbidities and mortality remain a global public health concern. Although preconception risk assessment has been found to enhance maternal, fetal, neonatal, and child health outcomes, few studies have explored preconception risks among premarital couples. The purpose of this study was to investigate the prevalence and predictors of preconception medical and behavioral risks among soon-to-be-married couples.

**Methods:**

A quantitative cross-sectional survey of 623 couples attending prenuptial meetings in rural and urban settings (churches and sector offices) was conducted from May to June 2024 using multistage cluster and purposive sampling. Data in the form of self-reported information were collected via structured interviewer-administered questionnaires. The Statistical Package for the Social Sciences (SPSS), version 29, was used to analyze the data.

**Results:**

Most participants (64%) were aged 21–30, with 81.5% from rural areas. Nearly half (49.3%) were classified as high-risk. Common medical risks included mental stress (46%), underweight (21.7%), use of teratogenic medications (16.1%), diabetes (12.5%), and hypertension (9.9%). Over 90% had never been screened for syphilis, hepatitis, anemia, or taken folic acid. Behavioral risks included inadequate nutrition (41%), heavy alcohol use (29%), use of non-prescribed/herbal medications, exposure to hazardous environments (20%), and inadequate physical activity. Males (OR = 1.28, p = .033) and urban residents (OR = 1.37, p = .011) had higher odds of risk. Shorter time until marriage was linked to increased risks (OR = 0.59, p < .001), while awareness of preconception care (OR = 0.09, p = .023) and medium-to-high income (OR = 0.79, p = .042) were associated with reduced risk.

**Conclusion:**

The overall prevalence of preconception risks among engaged couples was found to be high, indicating a need for targeted clinical and educational interventions for early prevention and management.

## Introduction and Background

Preconception risks refer to any conditions, diseases, lifestyle choices, or behaviors before pregnancy that can affect fertility, pregnancy outcomes, or fetal health [1,2] Preconception medical risks may include pre-existing chronic diseases such as diabetes, hypertension, asthma, and thyroid disorders; nutritional deficiencies like iron-deficiency anemia and folate deficiency; infectious diseases such as HIV, syphilis, hepatitis B/C, rubella, and toxoplasmosis; sexually transmitted infections (STIs) like chlamydia and gonorrhea; obesity or underweight; mental health conditions like depression and anxiety; genetic disorders; use of teratogenic medications; and dental issues [1,3].

Preconception behavioral and lifestyle risks may include smoking, alcohol consumption, drug use, poor diet (e.g., low intake of fruits, vegetables, and essential nutrients), physical inactivity, high caffeine intake, intimate partner violence, stress and poor sleep, environmental exposures (e.g., pesticides, radiation, and pollutants), and unprotected sex associated with STIs and unintended pregnancy [1,2].

Both medical and behavioral/lifestyle risks in the preconception period have been found to be associated with increased maternal and neonatal morbidity and mortality. These risks may lead to miscarriage, stillbirth, congenital anomalies/birth defects, preterm birth, and low birth weight [3,4]; maternal complications like pre-eclampsia, anemia, placenta previa, ectopic pregnancy, hydatidiform mole, or obstructed labor [5–8]; increased neonatal mortality due to poor fetal development; long-term child health and developmental issues; unintended pregnancies [9]; and mental health disorders affecting parenting and bonding [10–12].

Maternal and neonatal mortality and morbidity (MNMM) continue to pose a critical global health challenge [13,14]. Evidence indicates that nearly 800 women die every day due to pregnancy-related causes [15]. A staggering 95% of these deaths occur in low- and middle-income countries (LMICs), with Africa accounting for more than half of global maternal mortality and 70% of deaths occurring in the sub-Saharan Africa region [16]. In addition to maternal mortality and morbidity, LMICs face an estimated 1.4 million stillbirths and 3.6 million neonatal deaths within the first four weeks of life annually [17,18].

Although the burden remains high, literature shows that about 80% of pregnancy-related maternal and neonatal deaths and morbidities are preventable [19]. Evidence suggests that approximately 75% of maternal deaths and complications are linked to pre-pregnancy modifiable risk factors, including health conditions, attitudes, behaviors, and lifestyles of both partners [15,20–22]. Other studies show that 1.4 million stillbirths and 1.5 million neonatal deaths within the first week of life are associated with pre-pregnancy maternal or paternal health conditions and risky behaviors/lifestyles [9].

Certain behaviors, attitudes, and lifestyles adopted by either male or female partner before conception may induce complications in the prospective pregnancy. For example, studies show that paternal alcohol use prior to conception is correlated with a 28% increase in mortality one-year post-birth, a 23% higher likelihood of stillbirth, neonatal infections, and a 30% greater risk of congenital anomalies, preeclampsia, preterm birth, and infants born small for gestational age (SGA) [2,23]. Smoking before conception by either partner has been found to impact placental DNA methylation, which triggers pre-eclampsia, low birth weight, and preterm birth [24]. Additionally, 7–13.2% of low birth weight, preterm birth, and birth defects are linked to household and workplace air pollution, chemical toxicants from food or water, pesticide and solvent usage, and exposure to heavy metals such as lead and cadmium, along with organic solvents and herbicides [25,26].

Regarding pre-pregnancy medical conditions, studies show that untreated STIs, diabetes, hypertension, obesity, anemia, undernutrition, and use of teratogenic drugs are associated with poor pregnancy outcomes including miscarriage, ectopic pregnancy, hydatidiform mole, placenta previa, stillbirth, low birth weight (LBW), preterm birth, maternal preeclampsia, macrosomia, maternal and neonatal infections, neural tube defects, congenital heart malformations, congenital retinopathies, and physical disabilities in newborns [2,3,8,27–29]. Other studies show that preconception chronic stress and depression are associated with preterm birth, low birth weight, gestational diabetes mellitus (GDM), and postpartum depression [2,10].

The 2020 Rwanda Demographic and Health Survey (RDHS) reported a maternal mortality ratio of 203 per 100,000 live births and a neonatal mortality rate of 19 per 1,000 live births [30]. Various sources indicate that Rwanda’s reproductive-aged population faces a considerable burden of preconception risk factors. For example, the 2022 Rwanda Biomedical Center (RBC) report shows that 4.4% of this population experiences STI symptoms annually, 3.0% live with HIV [31], 19% of women are anemic, 7% are underweight, 8% are overweight, and 13.6% have unmet family planning needs [32]. Alcohol consumption is reported in 61.9% of men and 34.3% of women of reproductive age, with 12% smoking tobacco [33]. Risks of hypertension, diabetes, and other non-communicable diseases (NCDs) among this population have doubled in the last decade [34]. Furthermore, over 50% use over-the-counter drugs (OTCs) [35], while 50–80% use traditional medicines [36]. The United Nations Environment Programme (UNEP) reports that most reproductive-aged Rwandans are exposed to indoor air pollution and chemical toxicants due to biomass fuel use, pesticides, solvents, and heavy metals such as lead [37].

The preconception period has been identified as an optimal time to enhance pregnancy outcomes through the management of pre-existing conditions and adoption of healthy behaviors [38]. However, preconception care including risk assessment is still overlooked in many sub-Saharan African countries. In Rwanda, comprehensive preconception care is not yet systematically integrated into maternal health services. Emphasis is placed on antenatal, perinatal, and postnatal care, but these alone are insufficient to reduce maternal and neonatal morbidity and mortality. In practice, pregnancy risk assessment begins during antenatal care, yet reports show that over 90% of women attend their first visit late in the first trimester or after the embryonic stage has concluded [39]. At this stage, certain adverse outcomes such as congenital anomalies and maternal conditions can no longer be reversed or prevented. This undermines progress toward Sustainable Development Goal (SDG) Target 3.1 (reducing global maternal mortality to less than 70 per 100,000) and Target 3.2 (ending preventable newborn deaths and reducing neonatal mortality to at least 12 per 1,000 live births by 2030) [40].

Despite growing recognition of preconception care’s vital role in optimizing maternal, neonatal, and child health, services and data targeting reproductive-aged couples especially those preparing for marriage remain limited in LMICs. In Rwanda, 66% of couples live in formal/legal unions [41]. Before marriage, couples are required to attend education sessions on legal frameworks, theology, communication, sexuality, and family finance at churches and local administration sites (Ecole des fiancés/Betrothed schools) within 4–12 weeks before marriage. However, no known reproductive health education or risk screening services are offered. This is evidenced by the absence of reproductive health or preconception care content in the 2024 National Brides and Grooms-to-Be Education Protocol published by the Rwanda Ministry of Gender and Family Promotion (MIGEPROF) [42].

In many African contexts, the premarital period is a transitional phase enabling couples to shift toward procreation and family formation [43]. It is therefore essential to identify modifiable risk factors that, when managed early, can increase the likelihood of a healthy pregnancy and improve long-term outcomes for mothers, infants, and future generations [44].

Given the evident lack of research and limited data on preconception risk assessment in this specific subgroup of the reproductive-aged population, which may impede innovation and the development of effective initiatives, our study aimed to address these critical gaps. We investigated the prevalence and predictors of preconception medical and behavioral risks (including lifestyle) among soon-to-be-married couples.

Findings from our study can guide policymakers, healthcare providers, and researchers in crafting targeted, evidence-based preconception interventions especially for engaged, soon-to-be-married couples ultimately improving reproductive, maternal, and neonatal health and reducing morbidity and mortality in low-income settings.

## Methods

### Study design

This research employed a descriptive cross-sectional study design conducted from May to July 2024.

### Study population

The study focused on individuals in committed romantic relationships and who were preparing for formal civil or religious marriages in Rwanda. According to Presidential Decree No. 102/05, enacted on March 13, 1992, as was revised in 2024 the standard age for official marriage in Rwanda is set at 21 years [45]. The inclusion and exclusion criteria for participants were the following:

The inclusion criteria for participants were as follows: (1 Both male and female partners aged above 21 years, with males not exceeding 65 years and females not exceeding 48 years; (2) Participants must have been officially registered for either civil or religious marriage, with the marriage expected to take place within one to two months from the date of data collection; (3) The couple must self-identify as intending to have children after marriage. Then, exclusion included 1) old couples with male>65 and female exceeding child bearing age.2) Couples who do not intend to have children after marriage,3) Individuals in re-marriages or subsequent marriages,4) Couples where one or both partners were not willing to provide informed consent and 5) Those currently pregnant and who had attended antenatal care (ANC).

### Study settings

In Rwanda, couples attend premarital education sessions at least once a month for a period of three to six months before obtaining an official marriage license. These sessions are held at various venues, primarily sector headquarters and churches, commonly referred to as “École des fiancés” or the school of the betrothed.

Participants were recruited from these premarital counseling locations, including both sector headquarters and Écoles des fiancés. Although these venues are distributed across all thirty districts in both urban and rural areas, fifteen sites were randomly selected for this study.

The selected sites included: Western Province: Mushonyi, Gisenyi, Kanama, Southern Province: Huye (Huye District), Nyarusange (Muhanga District), Kamonyi (Kamonyi District), Northern Province: Muhoza (Musanze District), Ruli (Gakenke District), Shyorongi (Rulindo District), Eastern Province: Rwamagana (Rwamagana District), Kayonza (Kayonza District), Muhura (Gatsibo District) and Kigali City: Kinyinya (Gasabo District), Nyamirambo (Nyarugenge District), Masaka (Kicukiro District).

### Sampling techniques, sample size, and procedure

A total of 720 couples were initially screened from marriage prospect lists obtained from churches and local administration offices to reach the target sample size of 670, calculated using Cochran’s formula. After screening, 623 couples who met the inclusion criteria and agreed to participate were enrolled. Ultimately, 600 couples completed the study and were included in the analysis.

A three-stage multistage cluster sampling technique was employed: Stage 1: Fifteen districts across four provinces (Western, Eastern, Southern, and Kigali City) were selected using total sampling. Stage 2: Within each district, all sectors were enumerated, and two sectors were randomly selected by blindly drawing names from a basket containing all sector names. Stage 3: From each selected sector, 21 couples were purposively chosen. If a selected couple was found ineligible or declined participation, they were replaced by the next available eligible couple.

Both partners from each selected couple who met the inclusion criteria were interviewed using a structured questionnaire.

### Summary of sample size and sampling information flow chart

Refer to Fig 1.

**Fig 1:**
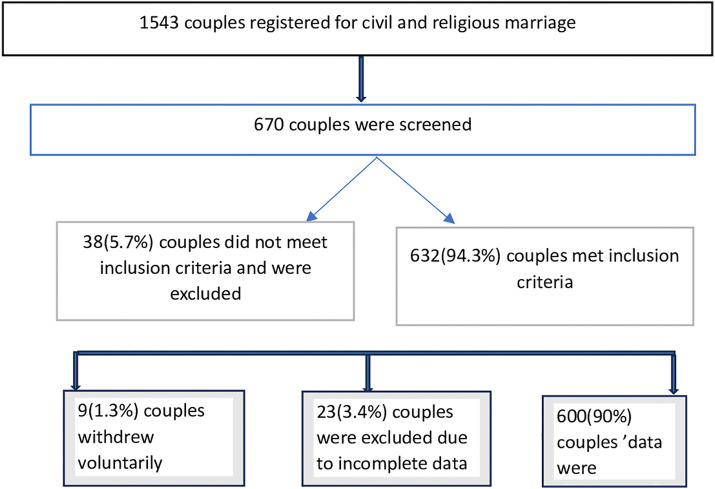
Participant recruitment and selection flow chart. Of 1,543 couples registered for marriage, 670 were screened; 38 were excluded for not meeting inclusion criteria, 9 withdrew voluntarily, and 23 had incomplete data. A total of 600 couples with complete data were analyzed. Rectangles indicate process stages, diamonds indicate exclusions or decisions, and arrows show participant flow. Percentages are based on the 670 couples screened.

### Data collection tool

Data collection for this study was conducted using a structured questionnaire (or checklist) administered through face-to-face interviews. The questionnaire was specifically designed based on the Preconception Health Risk Assessment Tool (PHAT) developed by the Center for Effective Practice (CEP) [46] the Preconception Health Assessment Tool by the International Federation of Gynecology and Obstetrics (FIGO) [47], and various other literature on preconception care.

During the development of this tool, careful consideration and customization were applied to reflect the local context and align with the study’s objectives. For instance, although certain high-tech tests were included in the prototype tools, they were excluded from this version due to limited accessibility. These excluded items included screenings for sickle cell anemia, thalassemia, Tay-Sachs disease, and vaccines not listed in Rwanda’s essential adult immunization schedule, such as Measles, Mumps, and Rubella (MMR). Similarly, not all medical conditions that could negatively impact health were incorporated into the risk checklist; instead, the focus was placed on conditions commonly diagnosed and treated in Rwandan health facilities.

Additionally, culture-specific practices that may influence preconception health and pregnancy outcomes were integrated. For example, considering the widespread use of over-the-counter drugs (OTCs) and traditional herbal remedies in Rwanda and their potential impact on conception and pregnancy items were added to assess the use of OTCs and uncontrolled traditional medicines. Given that traditional Rwandan diets rely heavily on staple foods often lacking sufficient micronutrients and high-quality proteins essential for reproductive health and fertility, questions were included to assess daily nutritional patterns based on locally available foods.

Cultural norms in Rwanda often discourage open discussions about sex, creating barriers for couples seeking preconception sexual health screening and increasing exposure to unintended pregnancies, HIV, hepatitis, and other sexually transmitted infections (STIs). Therefore, specific questions were added on condom use, sexual abstinence, pre-sex HIV/STI screening, and alcohol consumption before sex.

Because the majority of Rwandans rely on traditional farming methods with increased use of pesticides and herbicides and many work in informal jobs that expose them to pollutants under poor occupational safety conditions, additional items were included to capture exposure to harmful chemicals and toxic environments. These questions addressed the use of pesticides/herbicides, availability and use of protective equipment, and involvement in at-risk occupations.

The questionnaire was initially prepared in English and then translated into Kinyarwanda by a language expert. To ensure internal consistency, a third party translated the Kinyarwanda version back into English for verification.

The questionnaire consisted of four sections comprising a total of 42 items. The first section collected socio-demographic information, including age, sex, place of residence, education level, and monthly income. The second section explored relationship dynamics, such as the duration of the relationship, time remaining until marriage, and preferred timing for having children.

The third section contained 30 items divided into two parts. The first part assessed recent medical tests and health conditions, including infectious diseases (e.g., syphilis, HIV/AIDS), chronic illnesses (e.g., hypertension, diabetes), mental health status, and key blood tests (e.g., anemia, blood type). It also included questions on preconception vaccinations, such as hepatitis and tetanus.

The second part of this section, comprising 12 items, focused on behaviors and lifestyle factors. It addressed sexual risk behaviors, alcohol and drug use, tobacco smoking, nutritional habits, physical activity levels, and environmental exposures like exposure to pesticides, leads, air and water pollution, etc.

### Data quality control and measurements

To ensure high-quality research, meticulous data collection and monitoring procedures were implemented. A total of ten midwives and nurses were recruited as data collectors, supported by two public health specialists who served as supervisors during the data gathering phase. Prior to fieldwork, both data collectors and supervisors participated in a rigorous two-day training program. The training covered key topics including the study’s objectives, data collection methodologies, ethical considerations, and the use of the Kobo application on smartphones, tablets, and computers.

Before the main data collection began, a pilot study was conducted with 30 couples from a neighboring community that shared similar demographic characteristics with the target population but was not part of the study cohort. This pilot allowed for the identification and correction of ambiguities in questionnaire items, as well as the refinement of overlooked variables and grammatical errors to ensure clarity and precision.

Supervisors conducted daily checks to ensure data completeness and consistency. Any incomplete data entries were promptly excluded to preserve the integrity of the findings. The validity and reliability of the measurement instrument were thoroughly assessed prior to data collection. To ensure content validity, a panel of experts reviewed and refined each item. This panel included three senior midwives and nurses, one obstetrician, two reproductive health researchers, and one public health expert. The instrument demonstrated strong internal consistency, with a Cronbach’s alpha (α) value of 0.76 based on a sub-sample of participants (n = 30).

Data quality was further reinforced by employing research assistants who held bachelor’s degrees and were fluent in both local language Kinyarwanda and English.

The research measurements involved a systematic evaluation of participants’ responses to each risk item in the questionnaire. Responses were categorized as either “risk identified,” indicated by a “Yes,” or “risk not identified,” indicated by a “No.” Each identified risk was assigned a score of 1 point, while a score of 0 was allocated for instances where no risk was recognized. Consequently, the maximum achievable score across all items was 30, signifying that every item was classified as a risk.

At the conclusion of the assessment, individual scores were converted into percentages, with the highest possible score reflecting 100%. A cutoff point of 50% (15 out of 30) was established to classify participants into “low” and “high” risk categories. Participants who scored above the cutoff (>50% or >15/30) were categorized as high risk, meaning they had half or more of the identified risk factors. Those who scored below the cutoff (<50% or <15/30) were classified as low risk, indicating fewer than half of the risk factors.

The decision to use 50% as a threshold enabled a simplified binary classification that facilitated comparative analysis and interpretation of the data. Additionally, using 50% as a cutoff aligns with practices in similar epidemiological studies [48–50], where prevalence-based stratification is used to identify dominant patterns or trends. In public health research, prevalence at or near 50% is commonly employed as a threshold to dichotomize populations for risk assessment, allowing for practical and meaningful comparisons.

Dyadic couple risk was assessed by evaluating the individual risk scores of both partners within each couple. Couples were classified as “high-risk” if both partners independently fell within the high-risk category based on the established 50% cutoff stratification method. The percentage of high-risk couples was calculated using the total number of couples (n = 600) as the denominator.

### Data collection procedure

The data collection procedure began with obtaining signed informed consent from each participant, ensuring they fully understood the study’s purpose and their rights. Following consent, interviews were conducted in private settings by two trained research assistants to ensure comfort and confidentiality. Each assistant was assigned to interview participants based on gender one focused on male participants and the other on female participants. This approach fostered a relaxed atmosphere and encouraged open, honest responses.

During the interviews, participant responses were recorded using tablets or smartphones equipped with the Kobo Toolbox. This platform enabled real-time data processing, allowing for immediate calculation of each participant’s overall score based on their responses. The automated scoring system ensured that participants received instant feedback before leaving the interview site. To enhance data security, all responses were saved directly to the device upon entry and transferred to the collection server only after confirming secure storage.

Participants were comfortably seated in a conducive interview environment. Each partner within a couple was interviewed separately to promote open discussion. A shared identification code was used for both partners, supplemented by a letter “F” for females and “M” for males, to facilitate accurate and unified data entry for dyadic analysis. This systematic approach strengthened the reliability of data linkage between partners and ensured the integrity of the dataset throughout the process.

### Data management and analysis

Before commencing data analysis, an initial inspection was conducted to assess the consistency and completeness of the dataset and to identify any missing values or inconsistent responses (preliminary data cleaning). The data were then coded and entered into Excel before being exported to the Statistical Package for the Social Sciences (SPSS), version 29, for further cleaning and analysis.

Descriptive statistics were computed to evaluate frequencies, percentages, means, and standard deviations. Prior to conducting inferential statistical tests, the normality of continuous variables was rigorously assessed using the Shapiro-Wilk, Kolmogorov-Smirnov, and Anderson-Darling tests. This step was essential in determining the appropriate statistical methods for subsequent analyses.

Bivariate logistic regression analysis was performed to identify predictors of preconception risk among participants. A 95% confidence interval (CI) was applied, and a p-value of less than 0.05 (p < .05) was considered statistically significant.

### Ethical considerations

Ethical approval for this study was granted by the Institutional Review Board (IRB) of the College of Medicine and Health Sciences at the University of Rwanda, as documented in approval letter No. 337/CMHS-IRB/2024. Prior to data collection, the research team obtained permission from local authorities and church leaders at both district and sector levels.

Informed consent was obtained from each participant following a thorough explanation of the study’s purpose and procedures. Participants were informed of their right to withdraw from the study at any time without consequence. To protect confidentiality, a system of coded anonymity was employed. All interviews were conducted in private settings to ensure comfort and openness, and access to raw data was strictly limited to the principal investigator.

## Results

This study investigated the prevalence and predictors preconception medical and behavioral risks among engaged couples preparing for marriage.

### Respondents’ socio-demographic characteristics

Socio-demographic characteristics of participants are described in “Table 1**”.**

**Table 1:** Distribution of Socio-demographic characteristics among participants.

Socio-demographic characteristic of participants	Items	Frequency(n)	Percentage (%)
**Sex**	Female	600	50.0
	Male	600	50.0
**Age (in years and category)**	21–30 years old	776	64.7
	31–40 years old	346	28.8
	41 > years old	78	6.5
	µ = 29.5. SD=± 5.5	Maximum = 45	Minimum age = 21
**Education level**	Non-literate	53	4.4
	Primary	550	45.8
	Secondary	416	34.7
	TVET	78	6.5
	University	103	8.6
**Occupation**	Cultivator/Farmer	499	41.6
	Private sector servant	174	14.5
	Public sector	71	5.9
	Self-employed	213	17.8
	Not employed/student	199	16.6
**Religion**	Adventist	71	5.9
	Catholic	670	55.8
	Muslim	61	5.1
	Other	119	9.9
	Protestant	227	18.9
	Churchless	52	4.3
**Habitation**	Rural	978	81.5
	Urban	222	18.5
**Monthly income status**	From 200 000Rwf and <	726	60.5
	Above 200 000Rwf	474	39.5

***Notes****:* Individuals aged 21–35 are categorized as “Young age,” while those over 35 are considered “Advanced age.” Education below the secondary school level is labeled as “low educated,” whereas education ranging from secondary school to university level is referred to as “high educated.” VET stands for Technical and Vocational Education and Training.

“Table 1” presents the distribution of socio-demographic characteristics among study participants. The gender breakdown shows equal representation, with males and females each comprising 50% of the sample. The majority of participants (64.7%) were aged between 21 and 30 years, with a mean age of 29.5 years and a standard deviation of ±5.5. A significant proportion (45.8%) had attained only primary education, and 41.6% were engaged in agricultural work. Catholicism was the most common religious affiliation, representing 55.8% of the sample. Additionally, a considerable majority (81.5%) resided in rural areas. More than 60.5% of participants reported a monthly income of 200,000 Rwandan Francs (Rwf) or less.

### Overview of couples’ relationship and bonds patterns

“Table 2” describes the distribution of couples’ relationship and bonds overview among participants.

**Table 2:** Distribution of couples’ relationship and bonds overview.

Relationship bond information	Answer	Frequency(n)	Percent (%)
Length of Couples’ Relationships (Months)	*Short*	51	8.5
	*Medium*	392	65.3
	*Long*	157	21.7
	Mean, SD	µ = 22.6	SD=±16.3
Marriage remaining time*(months*)	Short time	536	89.3
	Long time	64	10.7
	Mean, SD	µ = 3.4	SD=± 1.2
Time the Child is Desired After Marriage (In Years)	Short time	536	89.3
	Long time	64	10.7
	Mean, SD	µ = 1.0	SD=±0.4
Couple partners live in the same geographical zone before marriage	No	338	56.33
	Yes	262	46.66

***Note.*** Relationship duration is classified as short (≤6 months), medium (≤24 months), and long (>24 months). Marriage duration is considered short if the remaining time is ≤ 3 months, and long if it exceeds 3 months. The timeframe in which a couple wishes to have a child is categorized as short if they desire a child within the first year of marriage, and long if their preference extends beyond the first year.

µ = Mean time; SD = Standard Deviation.

“Table 2” summarizes key metrics related to the relationships and bonding patterns of couples, including relationship duration, time remaining until marriage, interest in having children, and geographical proximity. Notably, 65.3% of couples reported a relationship length of 24 months or less, classified as medium duration. The average relationship duration (µ) was 22.6 months, with a standard deviation (SD) of ±16.3 months.

Regarding the time left until marriage, an overwhelming majority (89.3%) had three months or less remaining, indicating a short timeframe before marriage. The average time remaining until marriage (µ) was 3.4 months, with an SD of ±1.2 months.

All couples expressed a desire to have children, with 89.3% intending to do so within the first year of marriage a timeframe considered short. Additionally, more than half (56.3%) of participants resided in a different location from their partners.

### Preconception medical and health risks identified among couples preparing for marriage

Preconception medical and health risks identified among participants are presented in “Table 3”.

**Table 3:** Distribution of medical and health risks among participants.

Medical and general health status risks items		Males		Females		Total		Couple Dyadic risk (n, %)
		*Yes/At risk (n, %)*	*No/Not At risk (n, %)*	*Yes/At risk (n, %)*	*No/Not At risk (n, %)*	*Yes/At risk (n, %)*	*No/Not At risk (n, %)*	*Yes/At risk (n, %)*
Has High blood sugar		63 (5.25)	537 (44.75)	87 (7.25)	513 (42.75)	150 (12.50)	1050 (87.50)	39 (6.50)
Has Hypertension		47 (3.92)	553 (46.08)	68 (5.67)	532 (44.33)	115 (9.58)	1085 (90.42)	42 (7.00)
Has Chronic conditions (heart disease, liver, kidney, thyroid problem or asthma)		34 (2.8)	566 (47.17)	39 (3.25)	561 (46.75)	73 (6.08)	1127 (93.92)	11 (1.83)
Has dental problems		58 (4.83)	542 (45.17)	104 (8.67)	496 (41.33)	162 (13.50)	1038 (86.50)	50 (8.33)
Has Genetic a known problem		0 (0.00)	600 (50.00)	3 (0.25)	597 (49.75)	3 (0.25)	1197 (99.75)	0 (0.00)
Has a known anemia problem		0 (0.00)	600 (50.00)	0 (0.00)	600 (50.00)	0 (0.00)	1200 (100)	0 (0.00)
Body Mass index (BMI)	Underweight	186 (15.50)	414 (34.50)	75 (37.5)	525 (43.75)	261 (21.75)	939 (78.25)	111(18.50)
	Overweight	94 (7.83)	506 (42.17)	49 (24.5)	549 (45.75)	143 (11.92)	1057 (88.08)	38(6.33)
Has stress or mental health unwellness		245 (20.41)	355 (29.58)	307 (25.58)	293 (24.41)	552 (46.00)	648 (54.00)	190 (31.60)
Under teratogenic medication		84 (7.00)	516 (43.00)	109 (9.00)	499 (41.58)	193 (16.08)	1007 (83.92)	45 (7.50)
Not screened for HIV/AIDS		214 (17.8)	386 (32.17)	272 (22.66)	328 (27.33)	486 (40.50)	714 (59.50)	196 (32.66)
Not screened/ vaccinated for Hepatitis		555 (46.25)	45 (3.75)	539 (44.91)	61 (5.08)	1094 (91.17)	106 (8.83)	530 (88.33)
Not screened for syphilis and other STIs		584 (48.66)	16 (1.33)	574 (47.83)	26 (2.16)	1148 (95.67)	52 (4.33)	574 (95.67)
Did not receive tetanus vaccine shot		NA	NA	590 (98.33)	10 (0.83)	590 (98.33)	610 (50.83)	590 (98.33)
Not Tested for Blood type and Rh factor		542 (45.16)	58 (4.83)	558 (46.50)	42 (3.50)	1100 (91.66)	100 (8.33)	530 (88.33)
Not screened for anemia		566 (47.17)	34 (2.8)	561 (46.75)	39 (3.25)	1127 (93.91)	73 (6.08)	511 (85.16)
Not tested for Blood sugar		490 (40.83)	110 (9.17)	513 (42.75)	87 (7.25)	1003 (83.58)	197 (16.42)	484 (80.66)
Not checked for hypertension		503 (41.91)	97 (8.08)	509 (42.41)	91 (15.16)	1012 (84.33)	188 (15.67)	494 (82.33)
Did not take folic acid and other vitamins supplements		597 (49.75)	3 (0.25)	583 (48.58)	17 (1.41)	1180 (98.83)	20 (1.66)	572 (95.33)

**Note:**

• Body Mass Index (BMI) obtained by taking weight in kilograms (kg) divided by the square of height in meters (m2).

• Underweight if BMI of less than 18.5 kg/m2

• Overweight is if BMI of over than 25 kg/m2

• Sexually transmitted infections (STIs)

• NA = non-applicable

“Table 3” presents the distribution of medical and health risks among study participants. Approximately 46% reported experiencing poor mental health or stress. Unhealthy weight emerged as a dual risk factor, with 21.7% classified as underweight and 11.9% as overweight. Additionally, 16.1% of participants reported using teratogenic medications, while 13.5% experienced dental problems. Hypertension was present in nearly 10% of the sample, and 12.5% exhibited high blood sugar levels. Other chronic conditions including heart disease, thyroid disorders, and asthma were reported by 2.7% of participants.

Regarding preconception screening and health checkups, a substantial proportion of participants had not been screened for key health concerns: 40.5% had not undergone HIV testing, over 90% had not been screened for hepatitis, 95.7% had not been screened for syphilis, and 91.2% had not been tested for blood type and Rh factor. Furthermore, 83.6% had not been tested for blood sugar, and 84.3% had not been screened for hypertension. Notably, 95.3% of participants reported never taking folic acid, while over 93% of women had been screened for anemia.

Table 3 also presents findings related to dyadic medical and health risks among couples. Over one-third (31.6%) of couples reported that both partners experienced stress or mental health difficulties. In 7.5% of couples, both partners reported using teratogenic medications. Additionally, nearly 20% of couples had both partners classified as underweight, while 6.3% were categorized as overweight. Hypertension was present in both partners for 7.0% of couples, and 6.5% had both partners exhibiting high blood sugar levels.

Regarding testing and health checkups, 32.6% of couples had neither partner tested for HIV, while the majority (88.3%) had no partner screened for hepatitis. In 95.7% of couples, neither partner had been tested for syphilis, and in 88.3%, neither partner knew their blood type.

### Preconception behavioral and lifestyles risks identified among couples preparing for marriage

Preconception behavioral and lifestyles risks identified among participants are presented in “Table 4”.

**Table 4:** Distribution of behavioral and lifestyles risks among participants.

Preconception behavioral/lifestyle risks items		Males		Females		Total		Couple Dyadic risk (n, %)
		*Yes/At risk (n, %)*	*No/Not At risk (n, %)*	*Yes/At risk (n, %)*	*No/Not At risk (n, %)*	*Yes/At risk (n, %)*	*No/Not At risk (n, %)*	*Yes/At risk (n, %)*
Sexual behaviors risks	Unintended pregnancy	320 (26.67)	280 (23.33)	345 (28.75)	255 (21.25)	665 (55.42)	535 (44.58)	318(53.00)
	HIV	314 (26.17)	286 (23.83)	426 (35.50)	174 (14.50)	740 (61.67)	460 (38.33)	306(51.00)
	Syphilis	316 (26.33)	284 (23.67)	431 (35.92)	169 (14.08)	747 (62.25)	453 (37.75)	311(51.83)
	Hepatitis B&C	312 (26.00)	288 (24.00)	423 (35.25)	177 (14.75)	735 (61.25)	465 (38.75)	307(51.17)
Take heavy alcohol used		284(23.67)	316 (26.33)	72 (6.00)	528 (44.00)	356 (29.67)	844 (70.33)	53 (8.83)
Use drugs and caffeine use		24(2.00)	576 (48.00)	11 (0.92)	589 (49.8)	35 (2.92)	1165 (97.08)	9 (1.50)
Nicotine use/Tobacco		52(4.33)	548 (45.67)	19 (1.58)	581 (48.42)	71 (5.92)	1129 (94.08)	12 (2.00)
Use of non-prescribed medicines/herbal drugs		127(10.58)	473 (39.42)	222 (18.50)	378 (31.50)	349 (29.08)	851 (70.92)	107 (17.83)
Poor nutrition		278(23.17)	322 (26.83)	213 (17.75)	387 (32.25)	491 (40.92)	709 (59.08)	201 (33.50)
Poor physical activity		186(15.50)	414 (34.50)	75 (37.5)	525 (43.75)	261 (21.75)	939 (78.25)	111 (18.50)
Physical/sexual abuse		9(0.75)	591 (49.25)	187 (15.58)	413 (34.42)	196 (16.33)	1004 (83.67)	3 (0.50)
Exposure to harmful chemicals and toxic environment		182(15.17)	418 (34.83)	134 (11.17)	466 (38.83)	316 (26.33)	884 (73.67)	98 (16.33)

*Note:*

• Sexual risky behaviors were gathered through asking information about the individual practice of unprotected sexual intercourse, having multiple partners, inconsistent use of contraceptives, and engaging in sexual activities while under the influence of substances

• Poor nutrition means not taking a balance diet with a variety of fruits, vegetables, whole grains, proteins, and low-fat dairy products

• Poor physical activity means living in a sedentary lifestyle without exercising

• Harmful chemicals and toxic environment mean exposure to air, water and food pollution, use of pesticides, chemicals such as lead, smoke, and other toxic substances that may affect fertility health in living or working environment

• Heavy alcohol drinking means 4 or more drinks on any day or 8 or more per week,

“Table 4” presents the distribution of risky behaviors and lifestyle factors among engaged couples. The findings indicate that a significant number of participants engage in sexual behaviors that increase their risk of unintended pregnancy (55.4%), HIV exposure (61.67%), syphilis (62.2%), and hepatitis B and C infections (61.25%).

Regarding other preconception risk behaviors, 29.7% of participants reported heavy alcohol consumption, with 23.6% identifying as male. Additionally, 2.9% reported using drugs or caffeine, and 5.9% were smokers. Furthermore, 29.1% of participants reported taking non-prescribed medications or herbal products.

The results also reveal that 40.9% of respondents do not maintain a balanced and nutritious diet, while 21.7% reported living a sedentary lifestyle without regular physical activity. A concerning 16.3% of participants disclosed exposure to physical or sexual abuse, and 26.3% reported exposure to toxic environments in their daily living or working conditions.

### Overall preconception risk distribution among participants

**Fig 2:**
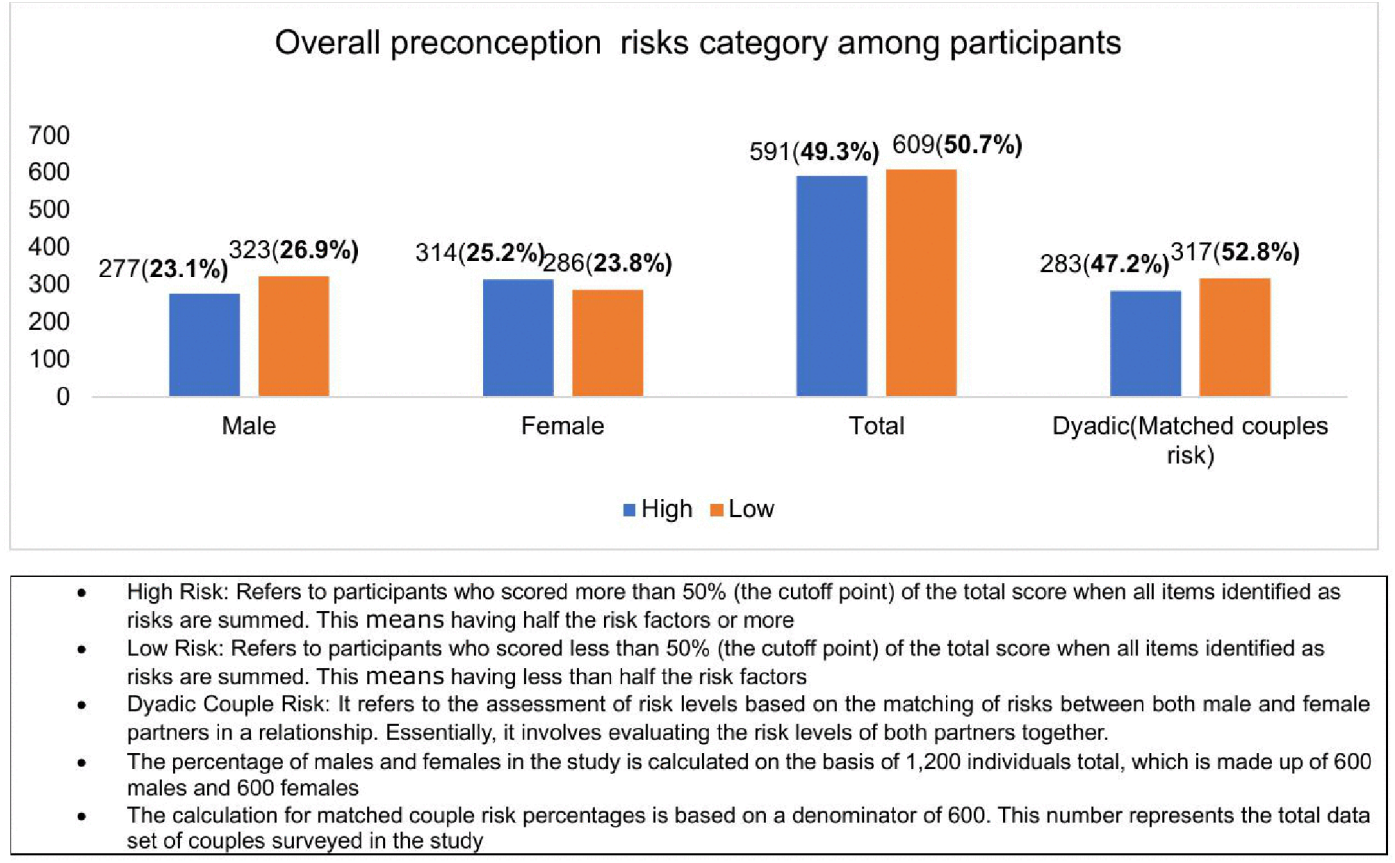
Distribution of preconception risk levels among participants. Risk levels are categorized as high or low across male, female, total, and dyadic (matched couple) participants. High-risk status is defined as a score exceeding 50% of the total risk score, indicating the presence of half or more of the identified risk factors. Low-risk status corresponds to scores below 50%. Dyadic risk represents the combined assessment of both partners within a couple. Percentages for male and female participants are based on 1,200 individuals (600 males and 600 females); dyadic risk percentages are derived from 600 matched couples. Bars representing high-risk participants are shown in red, and low-risk participants in blue. The x-axis denotes participant categories, and the y-axis indicates percentage distribution. Abbreviations: Dyadic risk = matched couple partners’ combined risk status; % = percentage.

As shown in Fig 2, approximately 49.3% of individual study participants were classified as high-risk, based on the presence of more than half of the concurrent risk factors outlined in the study’s predetermined checklist. Notably, females constituted a substantial portion of this group, representing 25.2% of the total high-risk population. Furthermore, among the 600 couples surveyed, 47.2% are found in dyadic or dual high-risk pairs, with both partners meeting the high-risk criteria.

### Determinants of preconception risks among participants

The determinants of medical and health risks preconception risks among participants are presented in “Table 5”.

**Table 5:** Predictors of preconception risks among participants.

*Independent Variable*		*Risk category*		*OR*	*95% CI*	*p*
		Low Risk n (%)	High Risk n (%)			
Gender	Females	323 (53.8)	277 (46.2)	1.28	1.03, 1.62	.033*
	Males	314 (52.3)	286 (47.7)	Ref	—	—
Age category	Young (21–35)	377 (42.0)	521 (58.0)	0.22	0.17, 0.29	<.001*
	Advanced (>35)	232 (76.8)	70 (23.2)	Ref	—	—
Education	Least educated (<secondary)	418 (49.1)	433 (50.9)	0.80	0.62, 1.03	.078
	High educated (≥ secondary)	191 (54.7)	158 (45.3)	Ref	—	—
Habitation	Rural	416 (53.3)	364 (46.7)	1.37	1.08, 1.73	.011*
	Urban	193 (46.0)	227 (54.0)	Ref	—	—
Religion affiliation	Christian	491 (50.7)	477 (49.3)	1.05	0.75, 1.34	.973
	Non-Christian	118 (50.9)	114 (49.1)	Ref	—	—
Employment status	Employed	427 (48.9)	446 (51.1)	0.90	0.69, 1.16	.412
	No job	182 (55.7)	145 (44.3)	Ref	—	—
Monthly income	Low income (≤ 200,000 RWF)	352 (48.5)	374 (51.5)	0.79	0.63, 1.02	.042*
	Medium to high income	257 (54.2)	217 (45.8)	Ref	—	—
Length of friendship	Short time (≤ 24 months)	352 (48.5)	343 (47.2)	1.25	0.97, 1.55	.086
	Long time (> 24 months)	226 (47.7)	248 (52.3)	Ref	—	—
Time left to marriage	Short time (≤ 3 months)	234 (43.7)	302 (56.3)	0.59	0.48, 0.75	<.001*
	Long time (> 3 months)	375 (56.5)	289 (43.5)	Ref	—	—
Time the child is desired	Immediately after marriage	279 (52.1)	257 (47.9)	1.09	0.88, 1.38	.418
	Later after marriage	330 (49.7)	334 (50.3)	Ref	—	—
Awareness/exposure to PCC info	No awareness/exposure	553 (48.6)	586 (51.4)	0.09	0.01, 0.72	.023*
	Has awareness/exposure	56 (91.8)	5 (8.2)	Ref	—	—

**Note:** OR = Odds Ratio; CI = Confidence Interval; Ref = Reference category; PCC = Preconception Care.

p < .05 (*), p < .001 (**); statistically significant predictors.

Awareness/exposure to PCC information = having heard about preconception preparedness from friends, media, or healthcare providers.

Being in high-risk category means having half the risk factors or more, then being in low-risk category means having less than half the risk factors.

“Table 5” illustrates the predictors of preconception risk based on bivariate logistic regression analysis. The results show that males were 1.28 times (or 28% more) likely to fall into the high preconception risk category (OR = 1.28, 95% CI [1.03, 1.62], p = .033). Participants of advanced age had approximately 78% lower odds of being in the high-risk group (OR = 0.22, 95% CI [0.17, 0.29], p < .001). Urban residents had 1.37-fold (or 37% higher) odds of being classified as high-risk compared to rural residents (OR = 1.37, 95% CI [1.08, 1.73], p = .011).

Income demonstrated a protective effect, with individuals earning a medium to high income having 21% lower odds of high preconception risk (OR = 0.79, 95% CI [0.63, 1.02], p = .042). A short time remaining until marriage was associated with increased risk, as those with a longer time to marriage showed a 41% reduction in the odds of being high-risk (OR = 0.59, 95% CI [0.48, 0.75], p < .001). Most notably, awareness and exposure to preconception care information reduced the odds of high risk by approximately 91% (OR = 0.092, 95% CI [0.01, 0.72], p = .023).

Other variables including education level, religion, employment status, length of friendship, and timing for childbearing were not statistically significant predictors of preconception risk.

## Discussion

The primary aim of this study was to investigate the prevalence and determinants of preconception medical and behavioral risks among engaged couples in Rwanda.

Our findings revealed a high overall prevalence of preconception risks, with nearly half (49.3%) of participants exhibiting multiple concurrent medical and behavioral risk factors (see Fig 2). This suggests that many couples may enter pregnancy with underlying health or behavioral issues that could compromise pregnancy outcomes. These risks may negatively affect both maternal and fetal health, potentially contributing to increased maternal and neonatal morbidity and mortality, as supported by Poix et al. [51].

Our results align with those of Dennis et al., who reported that 50% of participants were in the preconception risk category [52]. Similarly, a study conducted in Ethiopia found that the majority of participants were at risk during the preconception period [53]. In contrast, Robbins et al. in the USA reported a lower overall prevalence of 30% [1]. Specific risks such as unintended pregnancy and STIs were notably lower in their study (5.0%) compared to 55.4% in ours. Poor physical activity was reported in 58% of their participants, while over 83% of ours lacked sufficient physical activity. However, some risks were more prevalent in the U.S. study, for instance, cigarette smoking (21%) and obesity (38.6%) compared to 5.9% and 11.9%, respectively, in our population. These differences may be attributed to sociocultural factors and variations in healthcare system structures.

Most of the preconception risks identified in our study are common, preventable, and modifiable through individual behavior change and systemic healthcare improvements. For medical risks, we observed insufficient screening for infections such as HIV/AIDS, hepatitis, and syphilis, as well as for conditions like underweight, overweight, diabetes, hypertension, anemia, and blood type. These are manageable through routine health services. Additionally, mental stress, dental problems, and the use of teratogenic medications were prevalent but controllable risks.

Our findings are consistent with other studies. For example, Ukoha, et al. reported inadequate preconception screening across Sub-Saharan Africa [54]. Nekuei et al. in Iran found that only 3.4% of participants received physical examinations, and just 0.4% underwent routine lab tests for infections and chronic conditions [55]. In Ethiopia, Fetena et al. reported that 21.6% received preconception blood tests and physical exams [56]. These variations may reflect differences in cultural norms and healthcare infrastructure.

Behavioral risks were also prominent in our study: over 60% of participants were at risk for STIs and unintended pregnancies, 29.7% reported heavy alcohol consumption, 29.1% used non-prescribed medications or herbal products, 40.9% lacked a balanced diet, 21.7% lived sedentary lifestyles, and 26.3% were exposed to hazardous environments. These risks are preventable through early screening and health education. Dennis et al. similarly found that most preconception risks were preventable [52], and Poix & Elmusharaf’s multi-study analysis confirmed that preconception education and counseling significantly improved pregnancy outcomes [51].

Given the importance of addressing medical and behavioral risks prior to conception, our findings underscore the need for healthcare systems to prioritize preconception care for individuals in romantic relationships. Comprehensive screening and tailored interventions should be offered to both partners. Promoting healthy lifestyle choices through behavior change is equally essential.

Our study suggests that targeted health promotion strategies such as education on smoking, alcohol use, and nutrition can empower couples to make informed decisions. Community-based education and counseling initiatives can raise awareness about the importance of healthy lifestyles during the preconception period, ultimately improving pregnancy outcomes.

Regarding determinants of preconception risk, we found that certain subgroups were more vulnerable than others. Males were 28% more likely to be in the high-risk category, while older individuals had a 78% lower likelihood. Urban residents had 37% higher odds of elevated risk, whereas those with medium to high income had a 21% reduction in risk. A shorter time remaining until marriage was associated with increased risk, while those with longer time showed a 41% lower chance of being high-risk. Most notably, awareness and exposure to preconception care information reduced the odds of high risk by approximately 91%. These findings highlight the need for targeted interventions for specific subpopulations.

Although both partners were at risk, males were more frequently in the high-risk category, particularly for behavioral factors. Men were more likely to consume excessive alcohol, make poor dietary choices, avoid health screenings, experience inadequate sleep, and be exposed to harmful environments. These findings are consistent with Cassinelli et al., who reported that males in the preconception phase consumed alcohol 3–7 times per week, and 54.8% had poor sleep patterns [57]. This underscores the need for male-focused interventions to improve preconception health behaviors.

We also found that individuals exposed to preconception care (PCC) information were more likely to be in the low-risk category. Similar results were reported in Ethiopia [58], reinforcing the importance of educational programs in healthcare and the need for interventions that raise awareness among reproductive-aged populations.

Interestingly, employed participants had elevated risk, potentially due to limited healthcare access, psychosocial stress, and unhealthy lifestyle choices. Literature suggests that jobs with limited control over working hours may promote sedentary behavior and poor eating habits, negatively impacting health [59].

Furthermore, the short duration before marriage was associated with higher risk. According to the March of Dimes, the wedding preparation period can be stressful, contributing to unhealthy habits such as smoking, alcohol use, and poor diet [60]. Societal pressures and planning demands may also lead to irregular eating, increased consumption of convenience foods, and neglect of exercise. We recommend further research to explore these dynamics in greater depth.

Finally, we observed that different risks may intersect. For example, couples exposed to environmental toxins may also experience elevated stress, substance use, and limited access to preconception healthcare. While our study did not explore causal relationships in depth, we plan future research to investigate these intersections and provide a more holistic understanding of preconception risks.

### Strength and limitations of the study

Participants in this study were asked to self-report on sensitive health issues such as sexual behavior, alcohol consumption, and drug use, which may have introduced social desirability bias. Additionally, the study did not include laboratory testing or direct health measurements, which could have affected the accuracy of medical risk prevalence. We strongly recommend future research that incorporates clinical testing and screening to enhance the precision of risk assessment.

Due to the exploratory nature of the study and the absence of robust local data to determine the relative contribution of each risk factor, all preconception risks were treated with equal weight in the assessment. This approach was adopted to ensure simplicity and transparency in analysis.

The study was conducted in a country without a comprehensive preconception care program, which may have influenced the findings and limits their generalizability to populations with established preconception care services. However, this context also presents an opportunity to compare our results with settings that share similar healthcare infrastructure and cultural characteristics.

Importantly, this study provides valuable insights into preconception medical and behavioral risks, offering evidence to inform policymakers and stakeholders in reproductive and maternal health about critical areas requiring intervention.

Lastly, the study specifically focused on formally engaged couples preparing for marriage. While this focus is essential for understanding the unique risks faced by this subgroup, the findings cannot be extrapolated to other reproductive-age individuals in romantic relationships, such as those who are cohabiting or not officially engaged. We recommend further research to explore the dynamics and risks among other types of couples.

## Conclusion and recommendation

The high prevalence of preconception risks among soon-to-be-married couples highlights a critical public health concern. Most of the identified risks are both preventable and modifiable, underscoring the urgent need for targeted interventions. Our findings emphasize the importance of establishing comprehensive preconception care programs tailored to the health needs of individuals of childbearing age, particularly engaged couples.

Such programs should include systematic risk factor screening, education on healthy lifestyle choices, support for managing existing health conditions prior to conception, and expanded access to preconception counseling services. These interventions have the potential to significantly improve birth outcomes and enhance maternal, neonatal, and child health, as supported by existing research [51].

In addition, reproductive and public health efforts should prioritize raising awareness about the importance of preconception health, encouraging preventive measures even before pregnancies are planned, in line with recommendations from FIGO [47]. Integrating preconception health into broader reproductive health frameworks can promote a more holistic approach to health promotion.

Finally, further research is needed to evaluate the effectiveness of interventions designed to prevent and manage preconception risks. These studies will be essential for informing policy, refining program design, and improving reproductive health outcomes across diverse populations.

## Supporting information

S1 Fig Sample size and sampling information flow chart. This figure illustrates the flow of participant selection and sample size determination used in the study.
(TIFF)

S2 Fig Overall preconception risk distribution among participants. This figure presents the distribution of preconception risk levels across the study population.
(TIFF)

S1 File Minimal data set. This file contains the minimal data set necessary to replicate the statistical analyses and findings reported in this study.
(XLSX)
