# Supplementary figures and images for: Prevalence and predictors of preconception medical and behavioral risks among soon-to-be married couples: A quantitative cross-sectional survey in Rwanda
Source: PLoS One. 2026 Jan 16;21(1):e0336023. doi: 10.1371/journal.pone.0336023 (PMC12810831; doi:10.1371/journal.pone.0336023)

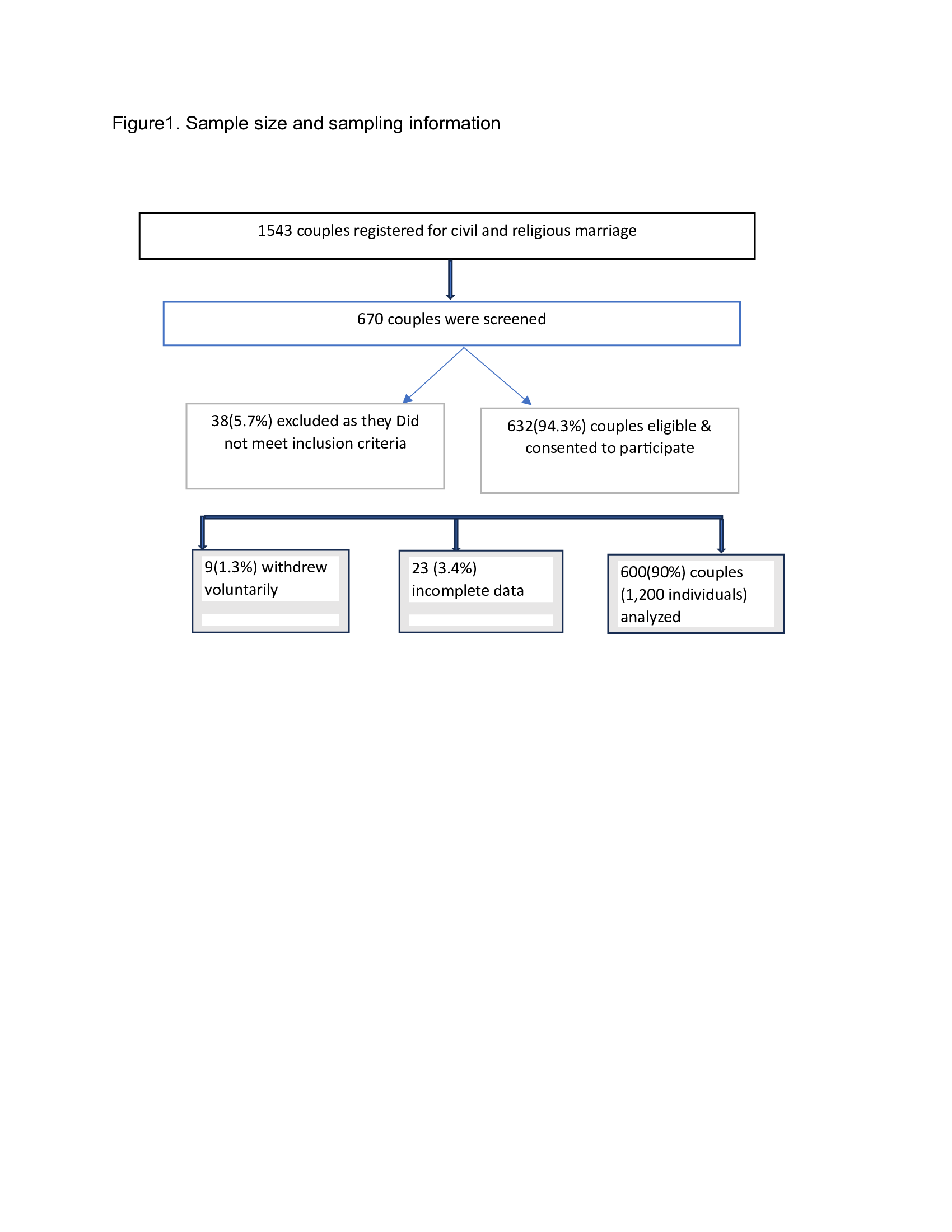

Supplement: S1 Fig — This figure illustrates the flow of participant selection and sample size determination used in the study. (TIFF) [file pone.0336023.s001.tiff]

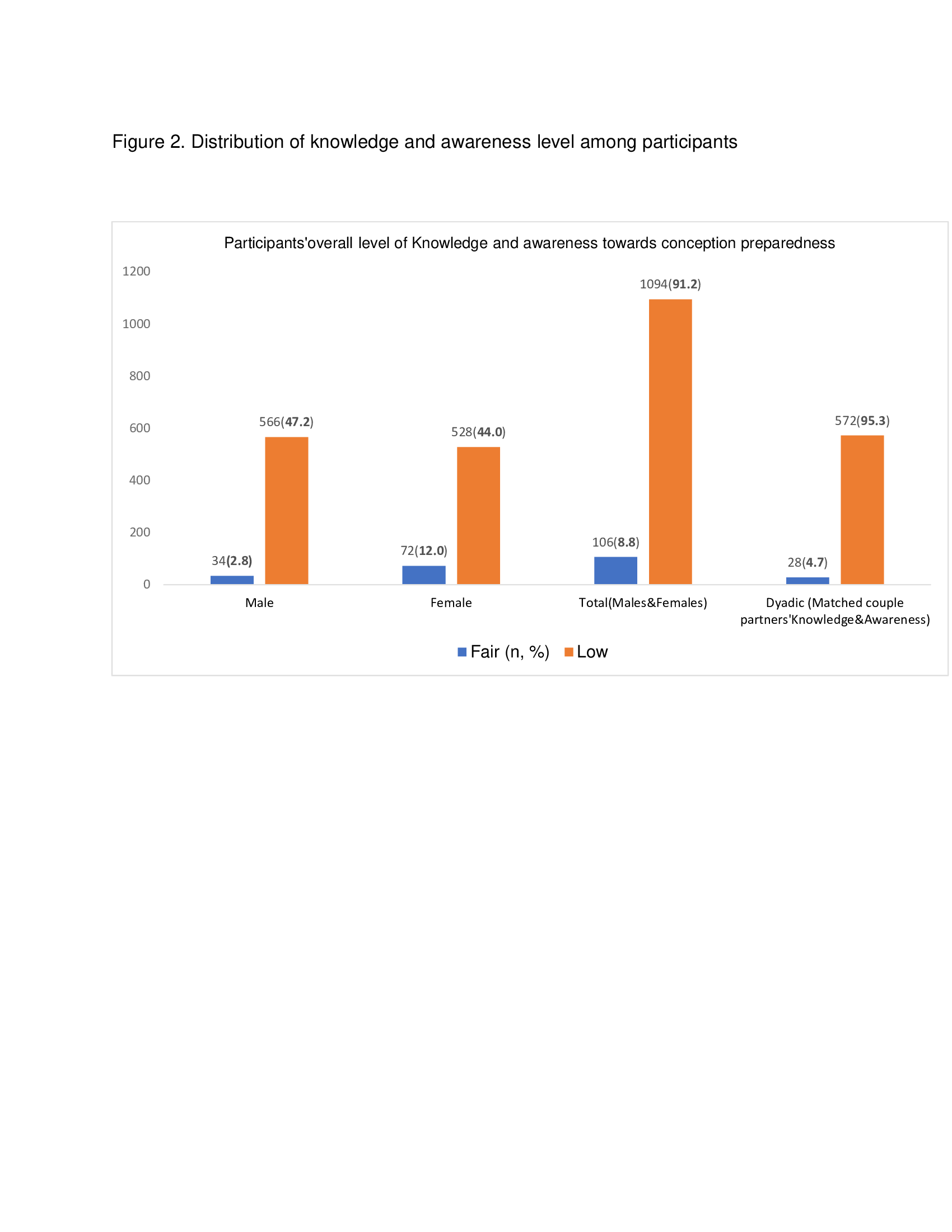

Supplement: S2 Fig — This figure presents the distribution of preconception risk levels across the study population. (TIFF) [file pone.0336023.s002.tiff]
